# IL-27 induces the expression of IDO and PD-L1 in human cancer cells

**DOI:** 10.18632/oncotarget.6530

**Published:** 2015-12-09

**Authors:** Grazia Carbotti, Gaia Barisione, Irma Airoldi, Delia Mezzanzanica, Marina Bagnoli, Simone Ferrero, Andrea Petretto, Marina Fabbi, Silvano Ferrini

**Affiliations:** ^1^ Department of Integrated Oncological Therapies, IRCCS AOU San Martino-IST Istituto Nazionale per la Ricerca sul Cancro, Genoa, Italy; ^2^ Laboratory of Oncology, Istituto Giannina Gaslini, Genoa, Italy; ^3^ Department of Experimental Oncology and Molecular Medicine, Fondazione IRCCS Istituto Nazionale dei Tumori, Milan, Italy; ^4^ Department of Surgery, Unit of Obstetrics and Gynaecology, IRCCS AOU San Martino-IST Istituto Nazionale per la Ricerca sul Cancro, and DINOGMI, University of Genoa Genoa, Italy; ^5^ Core Facilities, Istituto Giannina Gaslini, Genoa, Italy

**Keywords:** IL-27, IDO, PD-L1, STAT, Immunology and Microbiology Section, Immune response, Immunity

## Abstract

IL-27 is a member of the IL-12 family that is produced by macrophages and dendritic cells. IL-27 inhibits the growth and invasiveness of different cancers and therefore represents a potential anti-tumor agent. By contrast, it may exert immune-regulatory properties in different biological systems. We reported that IL-27 induces the expression of the IL-18 inhibitor IL-18BP, in human Epithelial Ovarian Cancer (EOC) cells, thus potentially limiting the immune response. Here, we tested whether IL-27 may modulate other immune-regulatory molecules involved in EOC progression, including Indoleamine 2,3-dioxygenase (IDO) and Programmed Death-Ligand (PD-L)1. IDO and PD-L1 were not constitutively expressed by EOC cells *in vitro*, but IL-27 increased their expression through STAT1 and STAT3 tyrosine phosphorylation. Differently, cells isolated from EOC ascites showed constitutive activation of STAT1 and STAT3 and IDO expression. These findings, together with the expression of IL-27 in scattered leukocytes in EOC ascites and tissues, suggest a potential role of IL-27 in immune-regulatory networks of EOC. In addition, IL-27 induced IDO or PD-L1 expression in monocytes and in human PC3 prostate and A549 lung cancer cells. A current paradigm in tumor immunology is that tumor cells may escape from immune control due to “adaptive resistance” mediated by T cell-secreted IFN-γ, which induces PD-L1 and IDO expression in tumor cells. Our present data indicate that also IL-27 has similar activities and suggest that the therapeutic use of IL-27 as anti-cancer agent may have dual effects, in some tumors.

## INTRODUCTION

Immune-regulatory mechanisms play a crucial role in cancer progression, as they limit the anti-tumor immune response [1, 2]. For example, the expression of Programmed Death-Ligand (PD-L)1 by cancer cells impairs T cell functions through the engagement of its receptor PD-1 at the T cell surface [3]. In addition, the enzyme Indoleamine 2,3-dioxygenase (IDO) is expressed in neoplastic cells and tumor-associated leukocytes or dendritic cells, and its expression correlates with a shorter survival, in different cancers [4]. IDO induces T cell dysfunction and apoptosis through the depletion of tryptophan and the generation of kynurenine [5].

Several experimental models and clinical studies in cancer patients indicate that targeting of immune-regulatory mechanisms may restore the immune response and mediate therapeutic effects. Recently, immune-enhancing monoclonal antibodies (mAbs) that target immune check-points such as anti-PD-1 and anti-PD-L1 have shown clinical benefit in different cancers [1, 2, 6, 7]. Although immunotherapy has been primarily proposed for “immunogenic” tumors, such as melanoma and renal cancer, anti-PD-L1 or anti-PD-1 blocking mAbs are currently being studied also in other cancers, including Epithelial Ovarian Cancer (EOC). Indeed, the immune system can recognize EOC cells [8, 9] because CTLs able to lyse EOC cells are present at the tumor site [10] and recognize different EOC-associated antigens [11]. Moreover, high tumor-infiltrating T cell counts have a positive impact on the clinical course of EOC [12-15].

We recently reported that IL-18 Binding Protein (IL-18BP), an inhibitor of the immune-enhancing cytokine IL-18 [16], is elevated in the serum and ascites of EOC patients [17]. IL-18BP is expressed in primary EOC and tumor-associated leukocytes *in vivo*, but not in human EOC cell lines, where it may be induced by stimulation with IFN-γ or IL-27. Thus, these cytokines may participate *in vivo* in an immune-regulatory network involving IL-18BP. Similar to IL-18BP, EOC cells express IDO *in vivo*, but not in culture, where it can be induced by IFN-γ stimulation [18]. In addition, IFN-γ mediates the induction of IDO and PD-L1 in both cancer and normal cells [19-21]. These effects may represent the outcome of a feed-back circuit, which counter-regulates the immune response and may lead to adaptive immune-resistance of tumors [1-3, 22, 23].

The possible role of IL-27 in immune-regulatory networks in cancer is still poorly understood [24, 25]. IL-27, a member of the IL-12 family, is a heterodimer, consisting of p28 (IL-27A) and EBV-induced gene 3 (EBI3) chains [24, 26]. IL-27 induces the proliferation of CD4+ T cells [27] and up-regulates IL-12R expression thus supporting Th1 polarization [28]. In addition, it directly inhibits growth or invasiveness of different cancer cells [29-34] and has immune-enhancing activity in different tumor models *in vivo* [35-38]. Altogether these findings support the potential use of IL-27 as anti-cancer agent [39]. However, IL-27 has anti-inflammatory and immune-regulatory functions and may limit some immune-mediated diseases [40-43]. For example, priming of naïve T cells in the presence of IL-27 converts them into PD-L1-expressing regulatory T cells, which limit IL-17 production [43].

To better characterize the role of IL-27 in regulating immune suppressive molecules in cancer, we investigated its ability to induce IDO and PD-L1 in EOC and other cancer cells.

## RESULTS

### IL-27 induces IDO expression in human EOC cell lines

**Figure 1:**
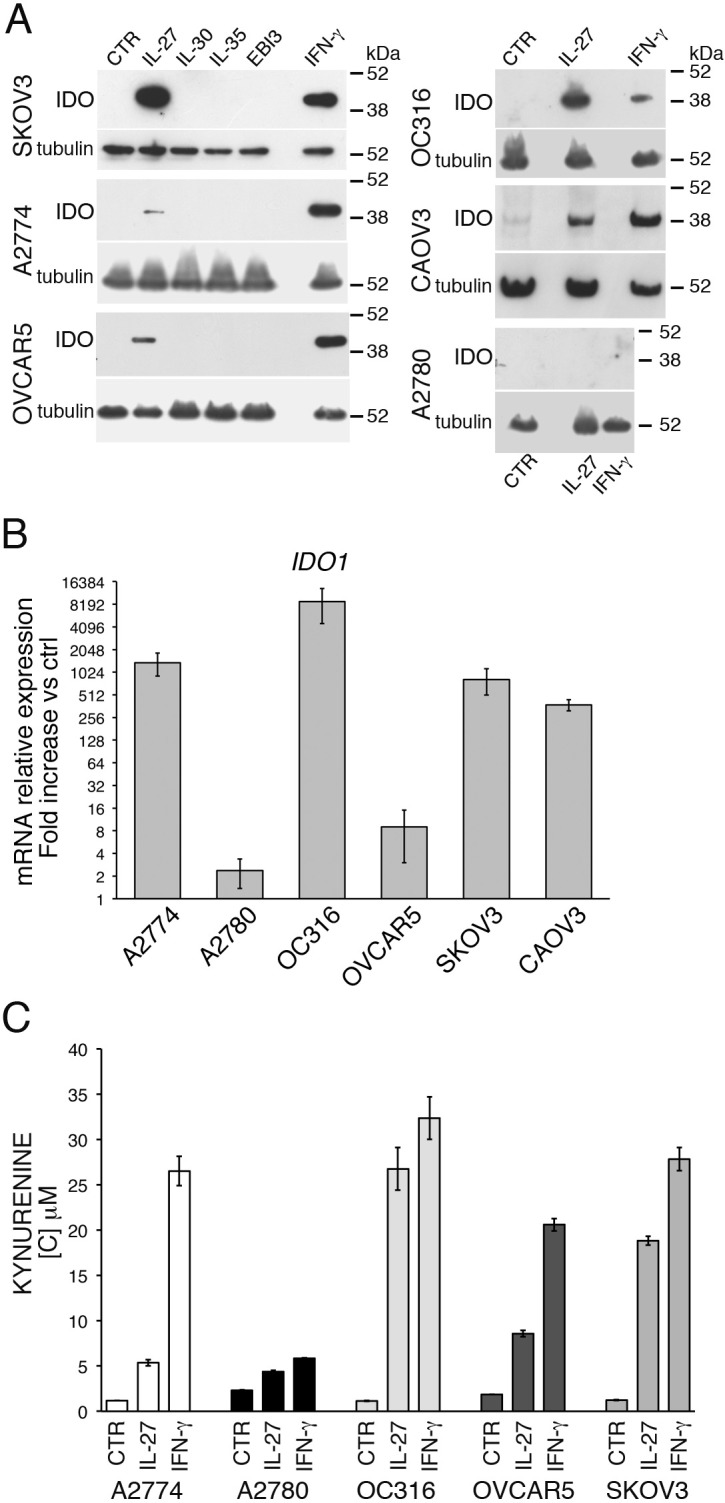
IL-27 induces IDO protein and mRNA expression in human EOC cells *in vitro* **A.** Western blot analysis of IDO expression in six EOC cell lines stimulated with the indicated cytokines or medium only (CTR) for 48 hours. α-tubulin is used as loading control. A representative experiment out of two is shown. **B.** QRT-PCR analysis of *IDO1* mRNA expression in EOC cells treated with IL-27 relative to untreated (Ctrl) cells. Data are the mean of three independent experiments and expressed as ΔΔCT-fold change. Error bars represent SD. **C.** Kynurenine production in the conditioned medium of IL-27, IFN-γ or untreated (CTR) EOC cells, as detected by HPLC analysis. Histograms represent mean values of three biological replicates and error bars are standard deviations.

IDO is involved in immune-suppressive circuits in EOC and several groups reported that it is expressed in EOC tissues [44-48]. As shown in Figure 1A, EOC cell lines do not constitutively express IDO protein, as detected by Western blot. However, if human A2774 EOC cells are implanted in SCID mice, IDO expression is observed in the engrafted tumor cells (Figure S1). These data suggest that factors produced within the tumor environment, different from murine IFN-γ, which is inactive on human cells, may induce IDO expression. As EOC cell lines are responsive to IL-27 [17], we asked whether IDO expression may be modulated by IL-27 in a panel of 6 cell lines representative of different sub-types of this cancer (Table S1) [49, 50]. As shown in Figure 1A both IFN-γ and IL-27 induce IDO protein expression in all the cell lines, with the exception of one (A2780). Accordingly, IL-27 strongly increases *IDO* mRNA expression from 9 to > 10,000-fold in all cell lines, but only marginally in A2780 cells (Figure 1B). Of note, IDO protein is enzymatically active as witnessed by the increase in kynurenine concentration in the conditioned media of IL-27- or IFN-γ-treated cells (Figure 1C), and, consistently, A2780 cells show minimal changes in kynurenine release upon cytokine stimulation. The inability of IL-27 to induce IDO expression in A2780 cells is not related to defective signaling, as our previous data showed that these cells respond to IL-27 by up-regulating STAT1 phosphorylation and IL-18BP mRNA and protein expression [17]. In addition, also IFN-γ failed to induce IDO expression in A2780 cells, suggesting that this cell line may have specific defects in IDO gene expression. Finally, we tested whether other members of the IL-12 cytokine family, sharing one of the two subunits of IL-27 (IL-27A or EBI3) may induce IDO in EOC cells. As shown in Figure 1A, IL-30, IL-35 or EBI3 failed to stimulate IDO expression in three IL-27-responsive cell lines. In addition, also the IFN-γ-inducing cytokine IL-12 and the GP130-signalling cytokines IL-6 and IL-11 failed to induce IDO expression (Figure S2) thus indicating that IDO induction in EOC cells is a unique feature of IL-27, among these cytokines.

### IL-27 up-regulates PD-L1 expression in human EOC cells

**Figure 2:**
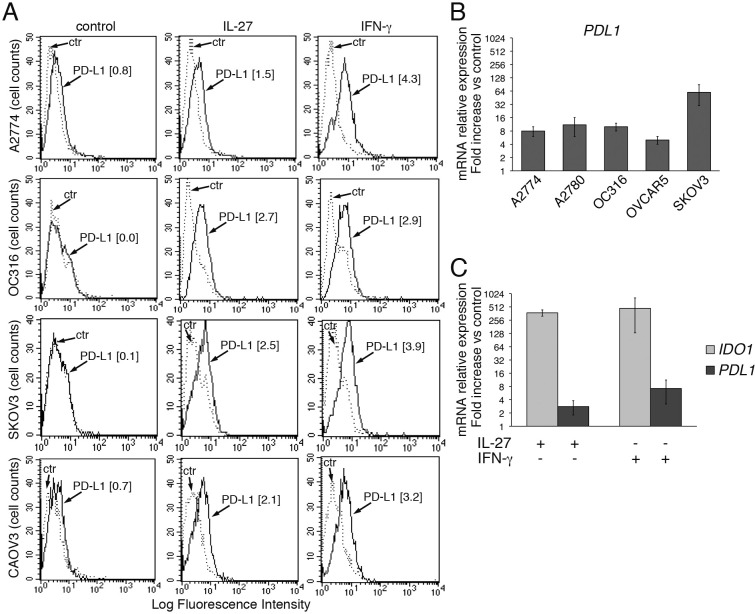
IL-27 increases PD-L1 surface protein and mRNA expression in EOC cells *in vitro* **A.** FACS analysis of surface PD-L1 expression in four EOC cell lines, cultured in the presence of medium (control), IL-27 or IFN-γ. Dotted lines are isotype-matched unrelated Ig staining controls. Numbers in brackets are Median Fluorescence Intensity (MFI) values calculated as median PD-L1 minus median Ig control. Data are representative of two independent experiments showing similar results. **B.** QRT-PCR analysis of *PDL1* mRNA expression in five IL-27-stimulated EOC cells relative to untreated cells. Data are the mean (±SD) of three independent experiments. **C.** Comparative analysis of *IDO1* and *PDL1* mRNA up-regulation by IL-27 or IFN-γ in a representative EOC cell line (CAOV3). Data are the mean of two independent replicates and are expressed as ΔΔCT-fold change. Error bars represent the minimum and maximum.

PD-L1 is a major cell surface immune-regulatory molecule in EOC [14, 51-54], as well as in other tumors, where it can be expressed either constitutively or in response to IFN-γ stimulation [20, 21]. As shown in Figure 2A, left panels, PD-L1 was not detected or was minimally expressed at the surface of EOC cell lines (control), as assessed by FACS analysis. However, both IFN-γ and IL-27 increased PD-L1 surface expression in 4 out of six cell lines analyzed (Figure 2A), while only minor induction was observed in the other two cell lines (not shown). In addition, IL-27 increased *PDL1* gene expression by 4 to 60-fold in the 6 EOC cell lines (Figure 2B and 2C), although this up-regulation was in general less evident than that of *IDO* gene (a representative EOC cell line, CAOV3, is shown in Figure 2C).

**Figure 3:**
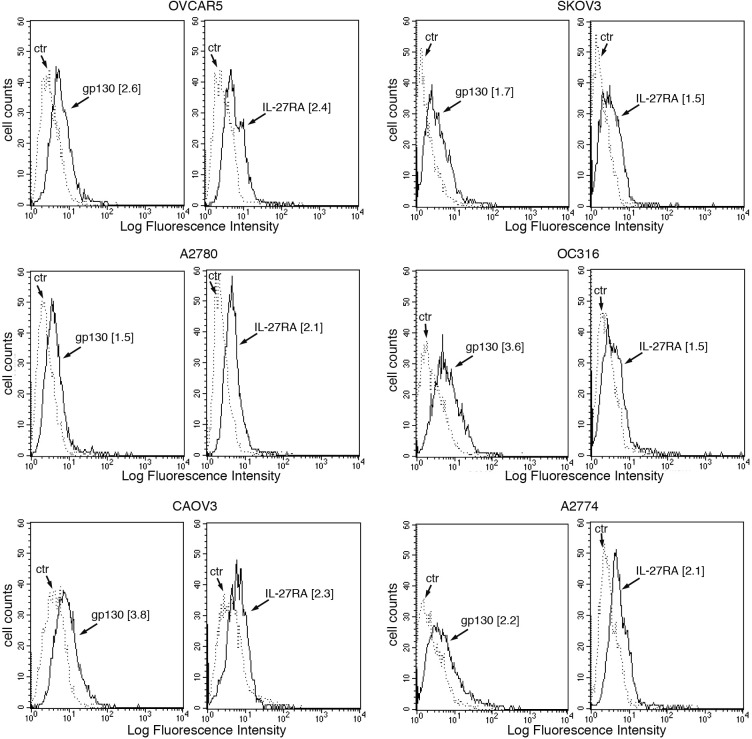
IL-27R chains GP130 and WSX1/IL-27RA are expressed in EOC cell lines FACS analysis of surface GP130 and WSX1/IL-27RA expression in six EOC cell lines. Dotted lines represent isotype-matched unrelated Ig staining controls. Numbers in brackets are MFI values calculated as median GP130 or WSX1/IL-27RA minus median Ig control. Data are representative of two independent experiments with similar results.

Altogether the biological activity of IL-27 in EOC cells suggested that it should act through the GP130/WSX1 receptor complex. Indeed, both GP130 and WSX1/IL-27RA receptor chains were detected in all cell lines by immunofluorescence (Figure 3).

We also asked whether IFN-γ and IL-27 could cooperate in inducing PD-L1 expression. A cooperative effect was indeed found both at sub-optimal (e.g. 100 IU/ml IFN-γ and 10 ng/ml of IL-27) and at high concentrations (1,000 IU/ml IFN-γ and 100 ng/ml of IL-27) (Figure S3).

### Role of STAT1 and STAT3 in IDO and PD-L1 regulation

**Figure 4:**
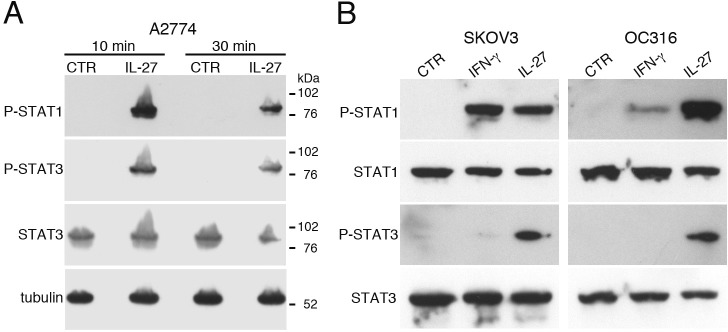
IL-27 induces STAT1 and STAT3 phosphorylation (P) in EOC cell lines *in vitro* **A.** Western blot analysis of tyrosine phosphorylated (P)-STAT1, P-STAT3 and STAT3 proteins in A2774 EOC cells either cultured for 10 or 30 minutes with medium (CTR) or with IL-27 (20 ng/ml). Total STAT3 and α-tubulin served as controls. Similar kinetics of STAT1/3 phosphorylation were observed in another cell line (A2780, not shown). **B.** Analysis of P-STAT1, STAT1, P-STAT3 and STAT3 proteins in SKOV3 and OC316 cell lines cultured in medium alone (CTR), or with IFN-γ or IL-27 (20 minutes). Total STAT1 and STAT3 were used as controls. Data are representative of three independent experiments.

**Figure 5:**
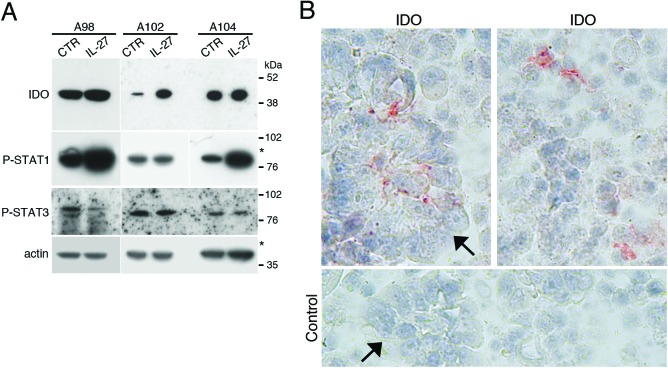
IDO and tyrosine-phosphorylated forms of STAT1 and STAT3 are constitutively present in cells from EOC ascites **A.** Western blot analysis of IDO, P-STAT1 and P-STAT3 proteins in neoplastic cells isolated from the ascites of three ovarian cancer patients cultured with medium (CTR) or IL-27 for 20 minutes. β-actin was used as loading control. * indicate lanes from a blot re-probed for IDO and phosphorylated STAT3 for which STAT1 and actin were presented in a previous article [17]. **B.** Immunohistochemical analysis of IDO expression in ascites cells. Both tumor cell nests (arrow) and scattered reactive cells express IDO protein.

IL-27 signals through STAT1 and STAT3 in different cell types [24]. IL-27 induced activation of both STAT1 and STAT3 through tyrosine phosphorylation also in EOC cells at 10-30 min after stimulation (Figure 4A and 4B). Moreover, cell populations, isolated from the ascites of three different patients, showed a constitutive phosphorylation/activation of STAT1 and STAT3 paralleled by IDO protein expression (Figure 5A). An additional control experiment showed the constitutive phosphorylation of STAT1 and STAT3 in cells isolated from ascites but not in a control, un-stimulated EOC cell line (Figure S4). By IHC, both tumor cell nests and scattered reactive cells contribute to IDO expression (Figure 5B). It is of note that immunohistochemical analysis of human ovarian cancer specimen revealed that IL-27-producing leukocytes are also present in some tumor microvessels (Figure S5). In these samples IDO is expressed in the microvessel wall and in some scattered cells in the stroma, while PD-L1 was found also in tumor cells (Figure S5A). These findings, together with our previous observation that leukocytes in the ascites and scattered in EOC tissues express IL-27 [17], support the concept that IL-27 released in tumor microenvironment may induce IDO expression.

**Figure 6:**
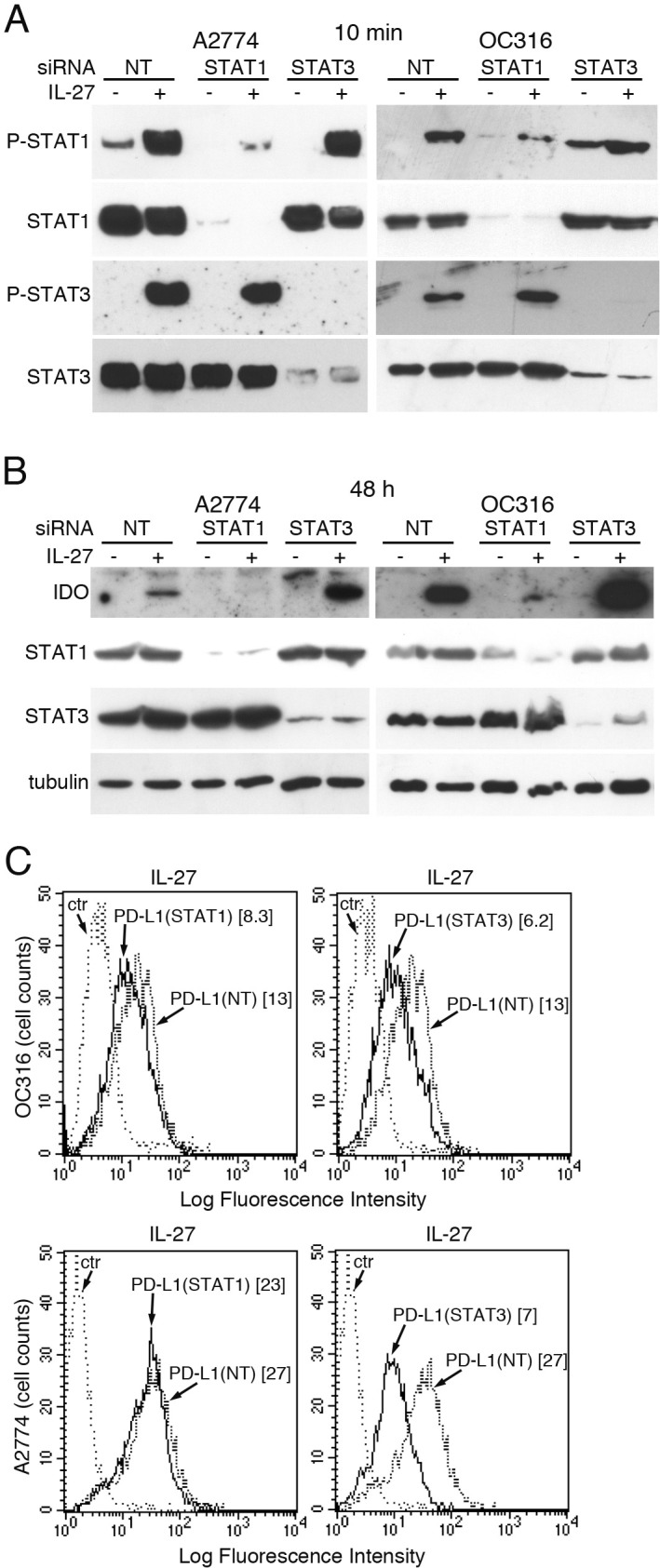
Silencing of STAT1 or STAT3 with siRNA effects IL-27-driven IDO or PD-L1 expression **A.** Western blot analysis of STAT1 and STAT3 unphosphorylated and phosphorylated (P) proteins in STAT1- or STAT3-silenced or scrambled (NT) siRNA-transfected A2774 and OC316 EOC cells untreated or treated for 20 minutes with IL-27 (20 ng/ml). Total STAT3 or STAT1 served as controls for STAT1- or STAT3-silenced cells, respectively. **B.** Western blot analysis of IDO expression in STAT1- or STAT3-silenced or scrambled siRNA-transfected A2774 and OC316 EOC cells untreated or treated for 48 hours with IL-27 (50 ng/ml). α-tubulin is shown as loading control. A replicate experiment is shown in Figure S6A **C.** FACS analysis of PD-L1 surface expression in STAT1- or STAT3-silenced or scrambled siRNA-transfected OC316 or A2774 EOC cells untreated or treated for 48 hours with IL-27 (50 ng/ml). Numbers in brackets are MFI values calculated as median PD-L1 minus median Ig control. The results of different experiments are shown in Figure S6B.

As both STAT1 [55, 56] and STAT3 [45, 57] have been involved in IDO and PD-L1 up-regulation in different cell types, we silenced STAT1 or STAT3, by specific siRNA, in A2774 and OC316 EOC cell lines, which are responsive to IL-27. Then, we treated these cells with IL-27 and analyzed them for IDO and PD-L1 expression. As expected, silencing of STAT1 strongly inhibited STAT1 expression and STAT1 phosphorylation induced by IL-27 treatment for 10 min, without effecting STAT3 expression and phosphorylation (Figure 6A). Conversely, STAT3 siRNA silencing had specific effects only on STAT3, but not on STAT1. Cells transfected with non-targeting siRNA were used as controls. Parallel cultures, stimulated with IL-27 for 48 h after silencing, showed a strong inhibition of IDO induction, in STAT1- but not STAT3-silenced cells, where it appeared even up-regulated (Figure 6B and Figure S6A). Conversely, STAT1 silencing failed to inhibit IL-27-induced surface PD-L1 expression, which was instead sensitive to STAT3 down-regulation, in both OC316 and A2774 cells (Figure 6C). Similar results showing that STAT3 silencing significantly inhibits IL-27-driven surface PD-L1 expression were obtained in replicate experiments (Figure S6B). Taken together, these results indicate that IL-27-driven up-regulation of IDO protein is predominantly STAT1-dependent, while PD-L1 is mainly regulated through STAT3 (Figure 6).

**Figure 7:**
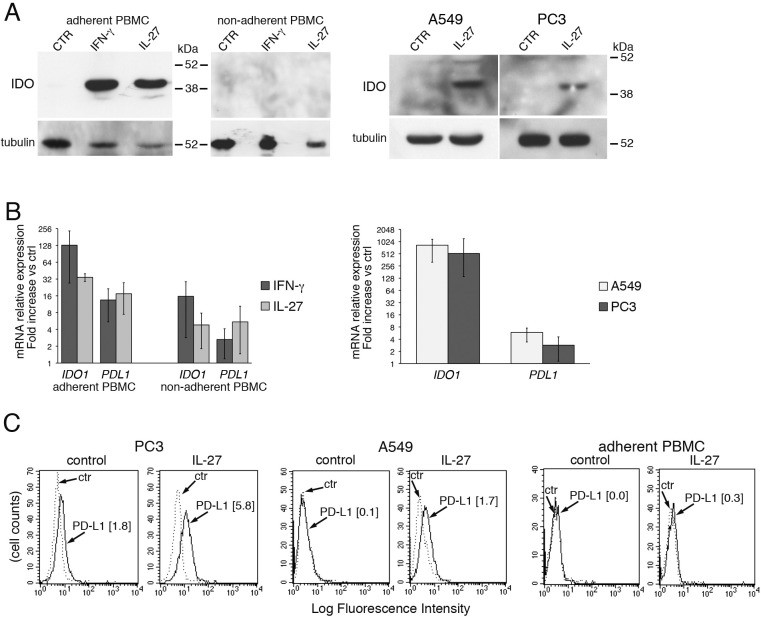
IL-27 induces PD-L1 and/or IDO expression in human PC3 prostate and A549 lung cancer cells and adherent PBMC **A.** Western blot analysis of IDO expression in human adherent PBMC, non-adherent PBMC and in PC3 and A549 cells treated with the indicated cytokines or medium only for 48 hours. α-tubulin is used as loading control. Similar results were observed in two additional experiments. **B.** QRT-PCR analysis of *IDO1* and *PDL1* mRNA expression in cytokine-stimulated adherent or non-adherent PBMC, PC3 and A549 cells relative to untreated cells. Data are expressed as ΔΔCT-fold change. Mean values of three independent experiments. Error bars represent SD. **C.** FACS analysis of surface PD-L1 in IL-27-treated or untreated PC3 and A549 cell lines and adherent PBMC. Dotted lines are isotype-matched unrelated Ig staining controls. Similar results were obtained in five different experiments (PC3: MFI 5.5 ± 2.7 *vs* 1.5 ± 0.6, mean ± SD in IL-27-treated *vs* un-stimulated cells, *P* = 0.03 by paired Student's *t* test; A549: MFI 2 ± 1 *vs* 0.43 ± 0.4, *P* = 0.01).

To verify whether the induction of IDO and PD-L1 expression by IL-27 was limited to EOC cells or was a common feature shared with other cancer cell types and normal cells, we tested PC3 prostate cancer cells and A549 lung adenocarcinoma cells, both of which were shown to respond to IL-27 stimulation, and human monocytes. Indeed, monocyte-derived tumor-associated macrophages play a fundamental role in the development and progression of cancer [58] and may express IDO protein in the tumor environment [59]. We found that monocyte populations, enriched by adherence to plastic from human PBMC, showed increased expression of IDO protein (a representative experiment out of three is shown in Figure 7A, left panels) and mRNA (Figure 7B, left) upon stimulation with IL-27 or with IFN-γ. Conversely, no significant induction of IDO was detected in the non-adherent cell fraction of the same PBMC (Figure 7A).

Finally, human prostatic PC3 and lung A549 carcinoma cells showed enhanced IDO mRNA and protein expression, in response to IL-27 stimulation (Figure 7B and 7A, respectively), although induction of IDO protein was in general lower than in EOC cells. IL-27 increased also PD-L1 mRNA (Figure 7B right) and surface protein expression in PC3 and at a lesser extent in A549 cells, while it induced virtually no changes in surface PD-L1 in monocytes (Figure 7C).

## DISCUSSION

Altogether these data show, for the first time, that IL-27 induces IDO and PD-L1 expression in human EOC cancer cells through activation/phosphorylation of STAT1 or STAT3, respectively. Among the IL-12 cytokine family, induction of IDO in EOC cells was a unique property of IL-27 as the related cytokines IL-12, IL-35, IL-30 and EBI3 failed to modulate such molecule. In addition, also IL-6 and IL-11, which signal trough GP130 were inactive. IL-27 also induced the expression of IDO and PD-L1 in human PC3 prostate cancer cells and in A549 lung adenocarcinoma and monocyte-enriched populations, suggesting a broader effect of IL-27. It should be noted that induction of IDO protein appeared low in PC3 cells, while PD-L1 induction was weak in A549 and very marginal in monocytes. The importance of PD-L1 expression in cancer cells as a major immune escape mechanism in the tumor microenvironment is supported by the clinical benefit of PD-L1 and PD-1 blocking antibodies [6, 7]. The current paradigm is that “adaptive” PD-L1 expression in tumor cells is induced by IFN-γ released during CTL recognition of tumor cells [20-22]. Our data suggest that IL-27, a cytokine produced by macrophages or DCs, may provide an alternative stimulus for PD-L1 expression, in cancer cells.

The expression of the two IL-27 chains, IL-27A and EBI3, in tumor-associated leukocytes in EOC ascites and tumor tissues suggested a role of endogenous IL-27 in the microenvironment of this tumor [17]. Nontheless, the assessment of the real role of IL-27 in ovarian cancer biology would require further studies in suitable syngeneic models. In agreement with the hypothesis of a potential role of IL-27 in EOC, tyrosine phosphorylated forms of STAT1 and STAT3 could be detected in cells isolated from the ascites, although the role of other STAT1/3 activating cytokines cannot be excluded. STAT1 signaling is essential for *IDO* transcriptional regulation by interferons [60]. However, recent reports indicate that autocrine induction of IDO by IL-6 is STAT3-dependent in some cancer cells or in myeloid suppressor cells [45, 61], suggesting that cytokines may induce IDO expression through different STAT pathways.

These data suggest that IL-27 may contribute to the generation of an immune-suppressive tumor environment, which could dampen the immune response through induction of IL-18BP, IDO and PD-L1, and add to the complexity of its effects in cancer. Indeed, several observations indicate that IL-27 has a dual role in cancer and in immune-regulation and inflammation [25, 40]. On one hand, IL-27 has been reported to display anti-inflammatory and immune suppressive effects. In fact, it induces expression of the ecto-ATPase CD39 on dendritic cells [62], IL-10 production [41, 63] and PD-L1 expression [43] in T cells and induction of Th1-like Treg cells [64]. These effects may limit autoimmune responses or anti-tumor immunity. Indeed, previous findings indicate that *Il27*−/− mice were more susceptible to experimental autoimmune encephalomyelitis than normal mice [42].

On the other hand, IL-27 has shown anti-tumor activity in different cancer models *in vitro* and *in vivo*, through different mechanisms, including direct effects on tumor cells or activation of an anti-tumor immune response [25]. For example, IL-27 directly inhibits proliferation and angiogenesis in B cell lymphoma, acute lymphoblastic leukemia, acute myeloid leukemia and multiple myeloma cells [65, 30, 29]. In addition, IL-27 inhibited *in vitro* and *in vivo* growth of prostate cancer cells and tumor angiogenesis [33] and also triggered anti-angiogenic responses through the induction of anti-angiogenic chemokines in mouse melanoma models [66]. More recently, IL-27 was shown to down-regulate stemness- and EMT-related genes in non-small cell lung cancer cells and to trigger myeloid cell anti-tumor activities in xenotransplant tumor models [67].

In conclusion, the net effect of IL-27 as an anti-tumor agent may depend on the balance of multiple factors. IL-27 similar to other cytokines studied as potential anti-cancer agents, e.g. IL-18 [68] or IL-21 [69], may have a dual role in immune-regulation. Indeed, our data provide new evidence that, beyond its well known anti-tumor effects, IL-27 may also elicit immune-regulatory circuits, particularly in the tumor environment, through the induction of PD-L1 and/or IDO in EOC cells and monocytes. Collectively these data suggest that the combination of IL-27 with inhibitors of IDO or PD-L1/PD-1 should enhance its anti-tumor effects and may be exploited in pre-clinical models of cancer immunotherapy.

## MATERIALS AND METHODS

### Ethics statement

Investigation has been conducted in accordance with the ethical standards and according to the Declaration of Helsinki and according to national and international guidelines and has been approved by the authors' Institutional Review Board.

Clinical samples and ascitic fluids were collected during surgical procedures before chemotherapeutic treatment from patients who gave written informed consent and used following the Institutional Review Board approval.

### Cells and cell treatments

The human EOC cell lines SKOV3 (ATTC), A2780 (ICLC), OC316 (ICLC), A2774 (IST Genoa), OVCAR5 and the lung carcinoma cells A549 (ATCC) were grown in RPMI 1640, with L-glutamine, 10% FCS and antibiotics (Lonza). The EOC cell line CAOV3 (INT Milan) was grown in DMEM with L-glutamine, Hepes, 10% FCS and antibiotics (Lonza). The prostate cancer cells PC3 (ICLC) were grown in HAM's F-12 with L-glutamine, 10% FCS and antibiotics (Lonza). A sample of each cell line was recently genotyped using the Cell ID™ System (Promega, G9500) and the GeneMapper® software, version 4.0.

Cells were seeded in 24-well plates in culture medium at 50×10^3^ cells/well or in 6-well plates at 150×10^3^ cells/well. The day after, culture medium was replaced with medium with or without human recombinant: IFN-γ (1,000 IU/ml, PeproTech, 300-02), IL-27, IL-12, IL-11, IL-6 (100 ng/ml, R&D System, 2526-IL-010, 219-IL-005, 218-IL-005, 206-IL-010), IL-35 (100 ng/ml, Enzo Life Sciences, ALX-522-140-C010), IL-30 (100 ng/ml, Abnova, H00246778-P01), EBI3 (100 ng/ml, Novus Biologicals, P4568).

For the analysis of IL-27-induced tyrosine-phosphorylated STAT proteins, 5×10^5^ EOC cells were incubated for 10 or 30 min at 37°C with or without 20 ng/ml of IL-27 in 0.5 ml of medium in a test tube. Cells were then rescued by centrifugation and immediately lysed.

PBMC were obtained from buffy coats by Lympholyte-H (Cedarlane, CL5020) density gradient centrifugation. After recovery and washings, PBMC were resuspended in RPMI 1640 supplemented with 4% AB serum, plated (10^7^ cells/2ml) into 6-well plates and incubated for 2 hours at 37°C in 5% CO_2_. Non-adherent cells were then removed and plated into new wells. Wells with adherent cells were supplemented with fresh medium. Both cell fractions were cultured for additional 48h in the presence or absence of IL-27 (100 ng/ml) or IFN-γ (1,000 IU/ml). Adherent cells consisted of >60% of CD14+ monocytes as detectect by FACS analysis with FITC-labelled anti-CD14 mAb (not shown).

### Western blot analysis

Cells, detached by 2 mM EDTA solution in PBS, were lysed in lysis buffer (20 mM Tris-HCl pH 7.4, 1 mM EDTA, 150 mM NaCl, 1% Brij97) containing 2 mM Na Orthovanadate and protease inhibitors (Roche Diagnostics, Complete Mini 04693124001). Lysates were resolved under reducing conditions by 10% SDS-PAGE and analyzed by Western blotting using the following antibodies: anti-IDO murine mAb (Upstate, clone 10.1, 05-840), rabbit anti-phospho-STAT1 (pY701) and anti-STAT1 anti-sera (Cell Signaling Technology, 9167 and 9172, respectively), murine anti-phospho-STAT3 (pY705) and anti-STAT3 mAbs (BD Transduction Laboratories, 612356 and 610190, respectively), and anti-β-actin or α-tubulin mAbs (Sigma-Aldrich, A2228 and T6074, respectively). Proteins were detected by ECL Prime (GE Healthcare, RPN2232) and autoradiography.

### RT-PCR analysis of IDO1 and PDL1 mRNA expression

Cells were detached by trypsin (Gibco, 12650-010), washed and total RNA was isolated by the NucleoSpin RNA kit (Macherey-Nagel, 740955.250) and reverse-transcribed using the SuperScript III Reverse Transcriptase (Invitrogen, 18064-071). Quantitative (Q)RT-PCR analysis was performed using the primers listed in Table S2. Amplification was carried out by the Mastercycler® ep realplex^4^ instrument (Eppendorf International) using the iQ™ SYBR^®^ Green Supermix system (Bio-Rad Laboratories, 170-8882). Relative quantification of mRNAs was calculated by the ΔΔCT method.

### Determination of kynurenine concentration

Conditioned media of IL-27 or IFN-γ-treated cells were collected, centrifuged, and tested for kynurenine. Samples were extracted with methanol and injected in a high resolution LC-MS/MS system, based on Orbitrap mass spectrometry. The MS/MS condition was used for a SRM quantitative method on kynurenine and the reference ion was the 94.07 m/z. Calibration curves, ranging from 1 μM to 50 μM, were run on two separate days and constructed from the peak-area of analyte resulting in a linear curve y = −257013+347555x with a R^2^ = 0.9906. The samples were prepared at least in duplicate and injected in triplicate.

### Immunofluorescence

Immunofluorescence with anti-GP130, anti-WSX1/IL-27RA-PE (R&D Systems, Clones 28126 and 191115), anti-PD-L1-PE or Isotype Control PE (eBioscience Bender, BMS-125983-41 and BMS-124724-41, respectively) was performed according to manufacturer's instructions. Cell fluorescence was analyzed in a FACScan (Becton&Dickinson) using the Cell Quest software. Gating on viable cells was performed using physical parameters and 10^4^ gated events were acquired.

### Small Interfering RNA (siRNA)-mediated STAT1 or STAT3 silencing

iBONI siRNA-Pool targeting different coding sequences of human STAT1 or STAT3 mRNA or Non-Targeting control siRNA pool (RIBOXX Life Science), were transfected in A2774 or OC316 cells using Lipofectamine 2000 Reagent (Invitrogen, 11668-019) in serum-free Opti-MEM medium (Gibco, 51985-026) according to manufacturer's instructions. After overnight incubation the medium was replaced with fresh standard culture medium. The following day, cells were stimulated with cytokines for different time intervals: 10-30 minutes for phosphorylated STAT detection or 48 hours for flow cytometry and Western blot analysis.

### Immunohistochemistry

Detection of IDO, PD-L1 and IL-27 by immunohistochemistry (IHC) was performed on sections of formaldehyde-fixed paraffin-embedded tumors explanted from mice or from 6 high grade serous EOC. Antigen-retrieval was performed with pH 6.0 10 mM citrate buffer (for PD-L1 and IL-27) or 1 mM EDTA, 0.05% tween20 pH 8.0 buffer (for IDO) in microwave oven. The sections were stained using rabbit anti-IL-27A antibody (LifeSpan BioSciences, LS-B2719-LSBio), anti-IDO mAb (Chemicon, clone 10.1, MAB5412) or rabbit anti-PD-L1 antibody (ProSci, 4059) overnight at 4°C. The antibody complex was revealed with the anti-rabbit or anti-mouse EnVision+ System-Peroxidase (Dako, K4002 and K4000, respectively) and 3-amino-9-ethylcarbazole (Sigma-Aldrich, AEC101-1KT). The sections were counterstained with modified Mayer's ematoxylin solution (Sigma-Aldrich, MHS16) and mounted in Fluoromount Aqueous Mounting Medium (Sigma-Aldrich, F4680). Images were captured with a Nikon Eclipse 80*i* light microscope equipped with a color camera, using a 40x objective.

## SUPPLEMENTARY MATERIAL FIGURES AND TABLES


