# Supplementary material for: IL-27 induces the expression of IDO and PD-L1 in human cancer cells
Source: Oncotarget. 2015 Dec 9;6(41):43267–80. doi: 10.18632/oncotarget.6530 (PMC4791231; doi:10.18632/oncotarget.6530)
Supplement: Supplementary file 1 [file oncotarget-06-43267-s001.pdf]

IL-27 induces the expression of IDO and PD-L1 in human cancer cells

Supplementary Material

Supplementary Table 1. Characteristics of the EOC cell lines used.

Name	Histotype	TP53	Other mutations
CaOv3	high-grade serous	mutated	
OVCAR5	high-grade serous	null	KRAS
SKOV3	atypical non-serous	wt*/null	ERB2 amplification, PIK3CA, ARID1A
A2774	endometroid	mutated	
A2780	mucinous	wt	PIK3CA, PTEN, BRAF, ARID1A
OC316	mucinous	wt	BRCA2, PIK3CA, BRAF, ARID1A

*discrepancies in the literature are reported. Histotypes are referred to recent molecular classifications, when applicable [49,50].

Supplementary Table 2. QRT-PCR primers.

Gene	Forward primer	Reverse primer
IDO1	GCCTGATCTCATAGAGTCTGGC	TGCATCCCAGAACTAGACGTGC
PDL1(CD274)	TGCCGACTACAAGCGAATTACTG	CTGCTTGTCCAGATGACTTCGG
GAPDH	GAAGGTGAAGGTCGGAGT	CATGGGTGGAATCATATTGGAA
POLR2A	GACAATGCAGAGAAGCTGG	GCAGGAAGACATCATCATCC

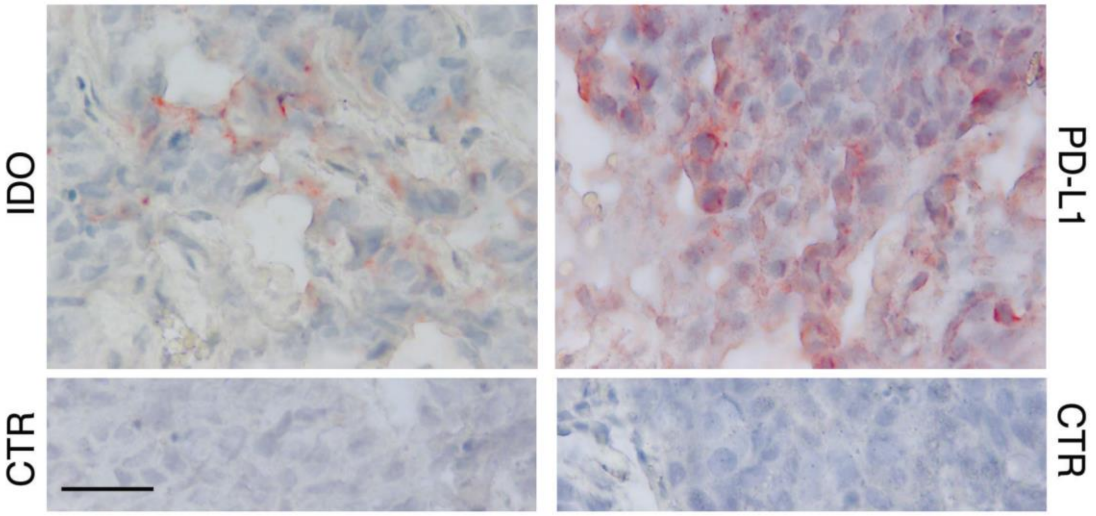

Supplementary Figure 1

Expression of IDO and PD-L1 in xenografts of the A2774 EOC cell line.

The experiments were performed according to the National Regulation on Animal Research Resources and approved by the Institutional Review Board. Six-weeks old NOD/SCID animals (bred in house) were injected i.p. with $5x10^6$ A2774 cells. Tumour masses were excised and fixed in 10% buffered formalin and processed for IHC as decribed in the Materials and Methods. Negative control (CTR) of a contiguous section is shown. Scale bar: 100 μm.

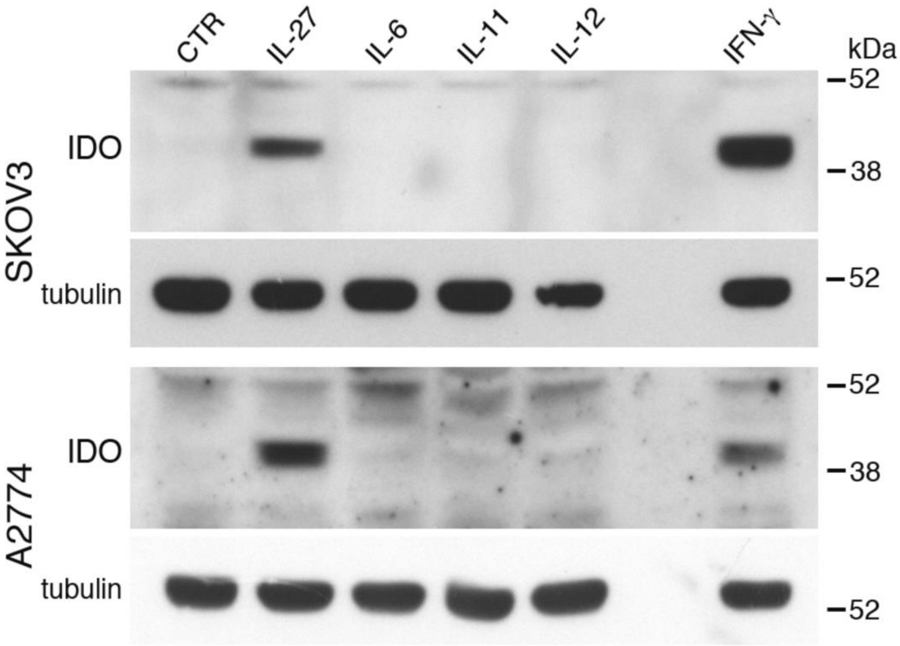

Supplementary Figure 2
IL-27 induces IDO protein expression in human EOC cells *in vitro*, while IL-6, IL-11 and IL-12 are inactive.

Western blot analysis of IDO expression in SKOV3 and A2774 EOC cell lines stimulated with the indicated cytokines (100 ng/ml) or medium only (CTR) for 48 h. α-tubulin is used as loading control.

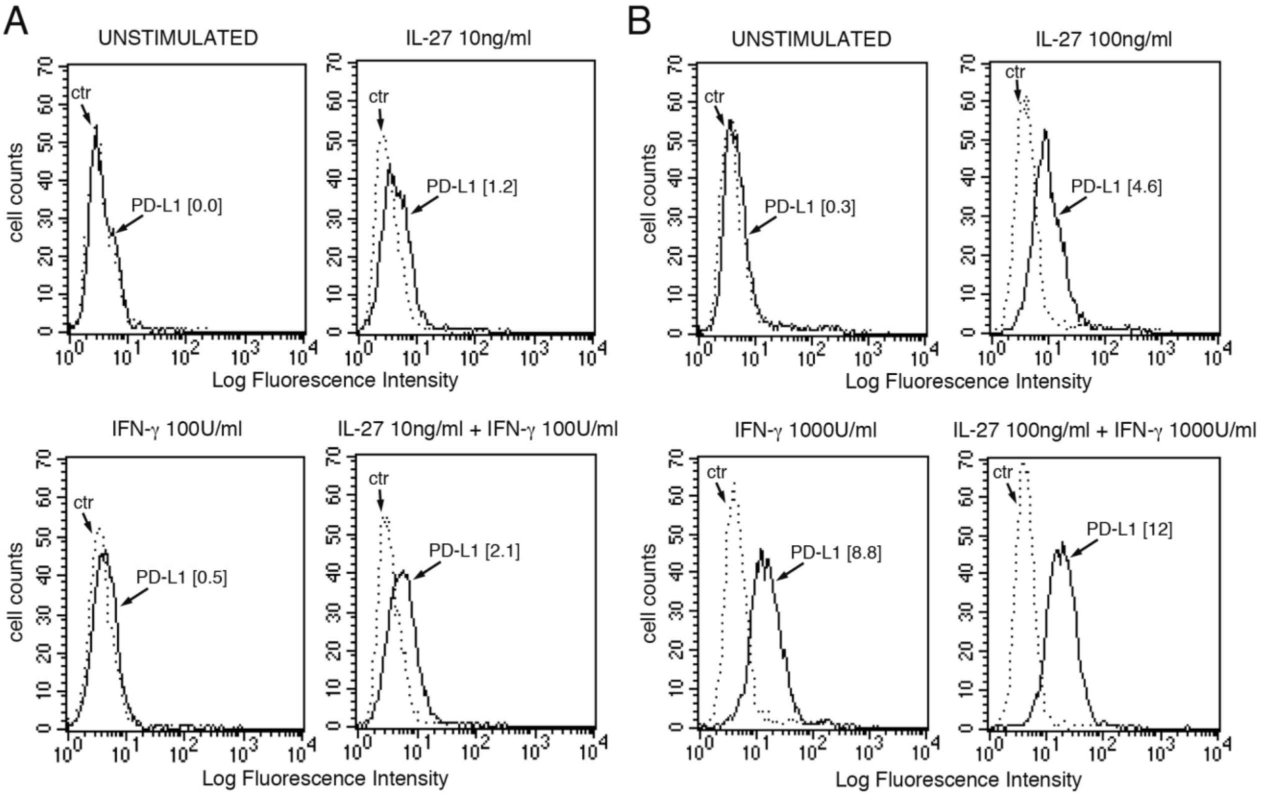

Supplementary Figure 3

IL-27 and IFN-γ cooperatively increase surface PD-L1 expression in EOC cells *in vitro.*

FACS analysis of surface PD-L1 expression in the SKOV3 EOC cell line, cultured in the presence of medium, IL-27 and/or IFN-γ (**A**: 10 ng/ml and 100 U/ml; **B**: 100 ng/ml and 1000 U/ml, respectively). Combination of the two cytokines showed additive effects. Dotted lines are isotype-matched unrelated Ig staining controls. Numbers in brackets are MFI values calculated as median PD-L1 minus median Ig control. A representative experiment out of three with similar results is shown.

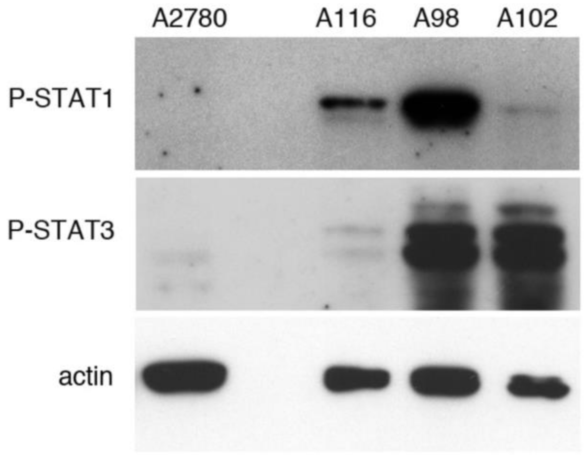

Supplementary Figure S4

STAT1 and STAT3 are constitutively phosphorylated in cells isolated from ascites but not in a representative cell line.

Western blot analysis of P-STAT1 and P-STAT3 proteins in neoplastic cells isolated from the ascites of ovarian cancer patients and in a negative EOC cell line (un-stimulated A2780), provided as control. β-actin was used as loading control. Constitutive STAT1 and STAT3 activation is observed in patient samples.

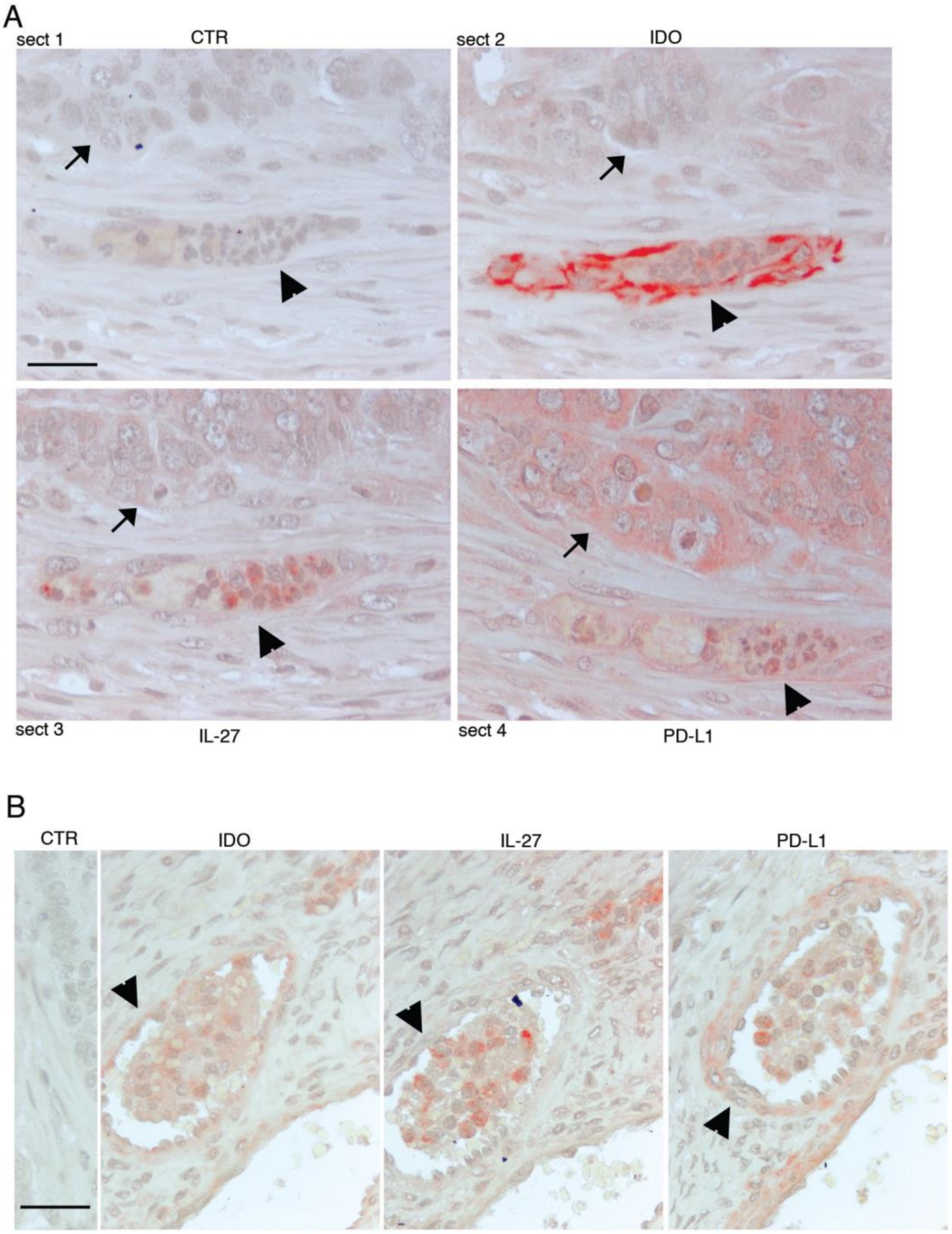

Supplementary Figure 5

Expression of IL-27, IDO and PD-L1 in human ovarian cancer specimen.

Immunohistochemical analysis of IL-27, IDO and PD-L1 expression in sequential sections of human ovarian cancer FFPE specimen. IL-27 is expressed by leukocytes within some tumor microvessels (arrowhead), and PD-L1 and IDO are expressed in neighbouring cells. Negative control (CTR) of a contiguous section is shown. Arrow indicates tumor cells. Scale bar: 100 μm.

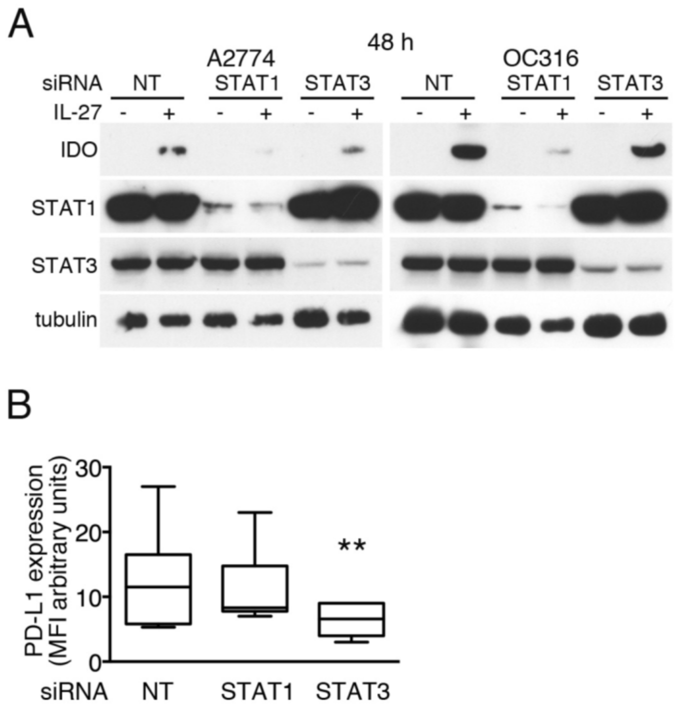

Supplementary Figure 6

Silencing of STAT1 or STAT3 with siRNA effects IL-27-driven IDO or PD-L1 expression (Replicate experiments).

A: Western blot analysis of IDO, STAT1 and STAT3 expression in STAT1- or STAT3-silenced or scrambled siRNA-transfected A2774 and OC316 EOC cells untreated or treated for 48 h with IL-27 (50 ng/ml). α-tubulin is shown as loading control. **B**: FACS analysis of PD-L1 surface expression in STAT1 or STAT3-silenced or scrambled siRNA-transfected (NT) OC316 and A2774 EOC cells untreated or treated for 48 h with IL-27 (100 ng/ml). Mean MFI values of six different experiments are shown as box-and-whiskers plot with minimum and maximum. MFI values were calculated as median PD-L1 minus median Ig control. Significant inhibition of IL-27-induced surface PD-L1 was observed only in STAT3-silenced cells (** P=0.03 by Wilcoxon matched-pairs signed rank test).
